# On Bark as an Antidote to Tartar-Emetic

**Published:** 1826-07

**Authors:** 


7. *Poisoning by Tartar-emetic.* Dr. Sauveton of Lyons, has lately published a case of this kind, where a lady swallowed, in a mistake, about a drachm of tartarized antimony dissolved in whey. In ten minutes after the accident, M. Sauveton was on the spot, and promptly exhibited about two ounces of tincture of yellow bark diluted in several glasses of cold water. The bad effects of the poison were almost entirely prevented, being limited to some nausea and epigastric pain, the latter continuing more or less for four or five weeks. He would advise the bark in substance to be given in such accidents in future, but we do not see any good reason for this preference—and the bulky nature of the powder would be more likely to increase the vomiting.

